# A New Method for Targeted and Sustained Induction of Type 2 Diabetes in Rodents

**DOI:** 10.1038/s41598-017-14114-4

**Published:** 2017-10-26

**Authors:** Dino Premilovac, Robert J. Gasperini, Sarah Sawyer, Adrian West, Michelle A. Keske, Bruce V. Taylor, Lisa Foa

**Affiliations:** 1 School of Medicine, University of Tasmania, Hobart, Tasmania Australia; 2 Menzies Institute for Medical Research, University of Tasmania, Hobart, Tasmania Australia; 3 Institute for Physical Activity and Nutrition (IPAN), School of Exercise and Nutrition Sciences, Deakin University, Geelong, Victoria Australia

## Abstract

Type 2 diabetes is a chronic metabolic disorder that is becoming a leading cause of morbidity and mortality. The prolonged time-course of human type 2 diabetes makes modelling of the disease difficult and additional animal models and methodologies are needed. The goal of this study was to develop and characterise a new method that allows controlled, targeted and sustained induction of discrete stages of type 2 diabetes in rodents. Using adult, male rats, we employed a three-week high fat-diet regimen and confirmed development of obesity-associated glucose intolerance, a key feature of human type 2 diabetes. Next, we utilised osmotic mini-pumps to infuse streptozotocin (STZ; doses ranging 80–200 mg/kg) over the course of 14-days to decrease insulin-producing capacity thus promoting hyperglycemia. Using this new approach, we demonstrate a dose-dependent effect of STZ on circulating glucose and insulin levels as well as glucose tolerance, while retaining a state of obesity. Importantly, we found that insulin secretion in response to a glucose load was present, but reduced in a dose-dependent manner by increasing STZ. In conclusion, we demonstrate a novel method that enables induction of discrete stages of type 2 diabetes in rodents that closely mirrors the different stages of type 2 diabetes in humans.

## Introduction

Among the different forms of diabetes, type 2 diabetes accounts for approximately 90% of cases and current estimates indicate that by 2040, approximately 642 million world-wide people will be living with type 2 diabetes^1,2^. This is likely to be a conservative estimate given that for every case of diagnosed type 2 diabetes, we know there is likely an additional, undiagnosed case^1,2^. This is most concerning in developing countries where as many as 50% of diabetics are undiagnosed and living with uncontrolled hyperglycemia^1,2^, the primary risk factor for development of cardiovascular, metabolic and neuronal disorders associated with the disease^3–6^. Therefore, development of animal models that accurately replicate the pathogenesis of human type 2 diabetes is of paramount importance and will enable identification of preventative and therapeutic strategies targeting both type 2 diabetes and associated pathologies.

Type 2 diabetes is a complex, chronic disorder and most people with type 2 diabetes progress through an early stage of obesity-associated insulin resistance prior to development of frank hyperglycemia^5,6^. With continuing insulin resistance, a compensatory increase in insulin production maintains normal circulating glucose concentrations^5–7^. This physiological shift impacts pancreatic beta cells early in disease progression and with time, leads to exhaustion of beta cell insulin production, clinically manifesting as frank type 2 diabetes^6^. The time-course of pre-clinical and then clinical type 2 diabetes progression can be incredibly long and thus makes modelling discrete stages of type 2 diabetes incredibly difficult.

With the exception of genetic models, which are costly and do not accurately model human type 2 diabetes, the most commonly used animal model of type 2 diabetes is the high fat diet (HFD) fed rodent injected with streptozotocin (STZ)^8–12^. This model accelerates the time-course of type 2 diabetes by initiating a state of obesity-associated insulin resistance using a period of HFD feeding^7,13^, followed by an injection of STZ to deplete pancreatic beta cells^8,14^. A few days after STZ administration, these animals develop severe hyperglycemia in the near absence of insulin production, exhibit marked weight loss and have short life expectancy^15^. These physiological changes currently confine the HFD + STZ model to investigation of end-stage type 2 diabetes, which more closely resembles type 1 diabetes^16,17^. Some studies have utilised multiple low-dose STZ injections and argue that this approach enables better control of the ensuing hyperglycemia based on the notion that smaller, repeated insults impart greater control over the extent of beta cell death^18,19^. However, while leading to short-term hyperglycemia, long-term studies (≥four weeks) are confounded by the ability of the remaining beta cells to compensate/regenerate resulting in a reversion toward normoglycemia^20,21^. This is particularly evident in younger rodents and suggests that the efficacy of injection methods may also be markedly influenced by the age at which STZ is injected^20,21^. Given these limitations, the aim of the current study was to develop a new animal model that allows targeted and sustained induction of discrete stages of type 2 diabetes where animals remain obese, exhibit mild-moderate hyperglycemia and retain the ability to produce insulin, or those that exhibit frank type 2 diabetes.

## Results

### STZ is stable over a 14-day period

**Figure 1:**
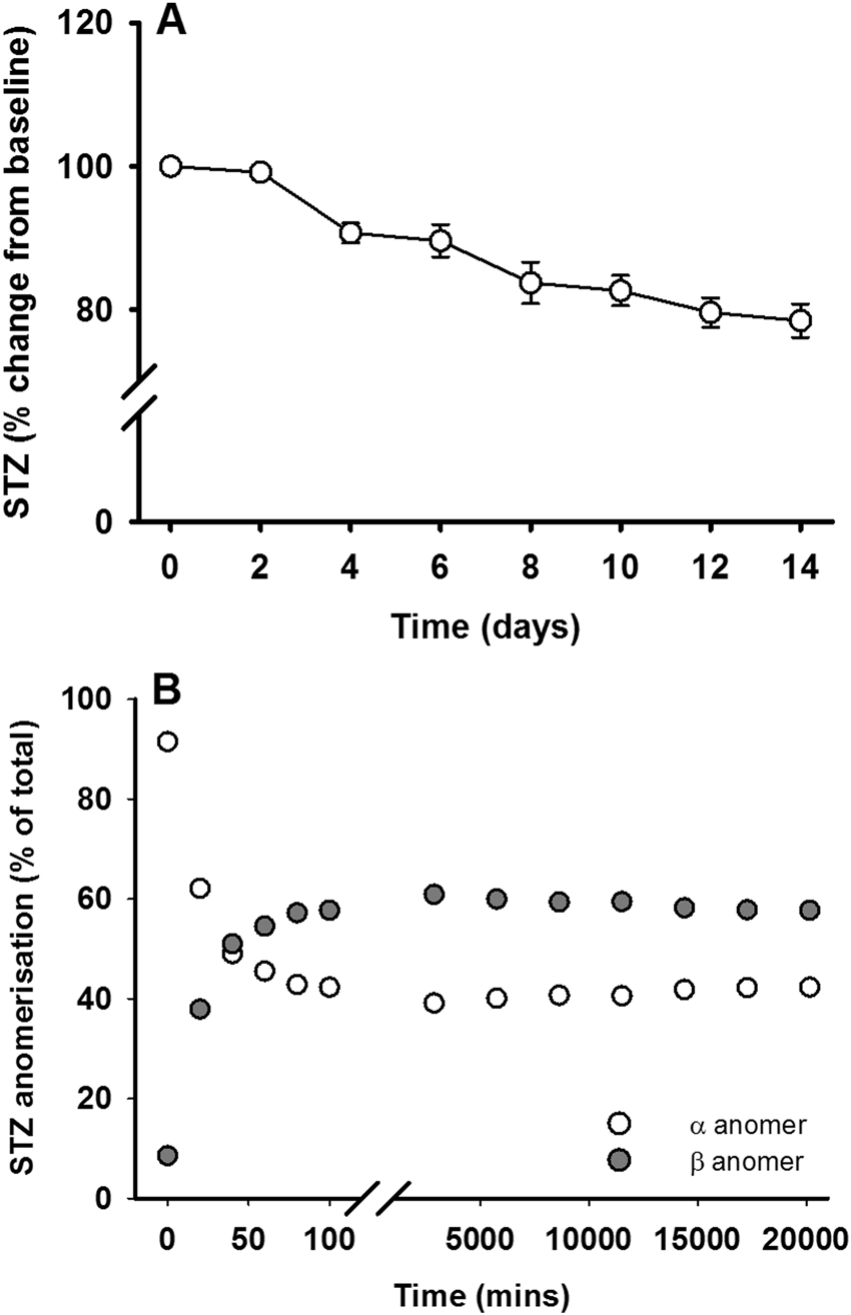
Streptozotocin is stable over 14 days. (**A**) The total concentration of STZ was determined every two days from dissolution in citrate buffer using HPLC and plotted as percent change from day 0 (representing 100%). (**B**) The relative amount of each STZ anomer was also monitored using HPLC over the first 100 min post-dissolution and then every two days thereafter. Data are means ± SEM for n = 4 independent solutions of STZ.

Due to the slow release of STZ from the mini-pump, we first investigated the stability of STZ in citrate buffer across 14-days (delivery period of the mini-pump) at 37 °C. Over the course of two-weeks, STZ degradation was approximately 20% (Fig. 1A). The ratio of alpha/beta STZ anomers reached equilibrium (43/57%, respectively) within the first two hours of dissolution and remained constant across the two-week period (Fig. 1B).

### Rats given a HFD and osmotic mini-pump-infused STZ exhibited increased body weight and adiposity

**Figure 2:**
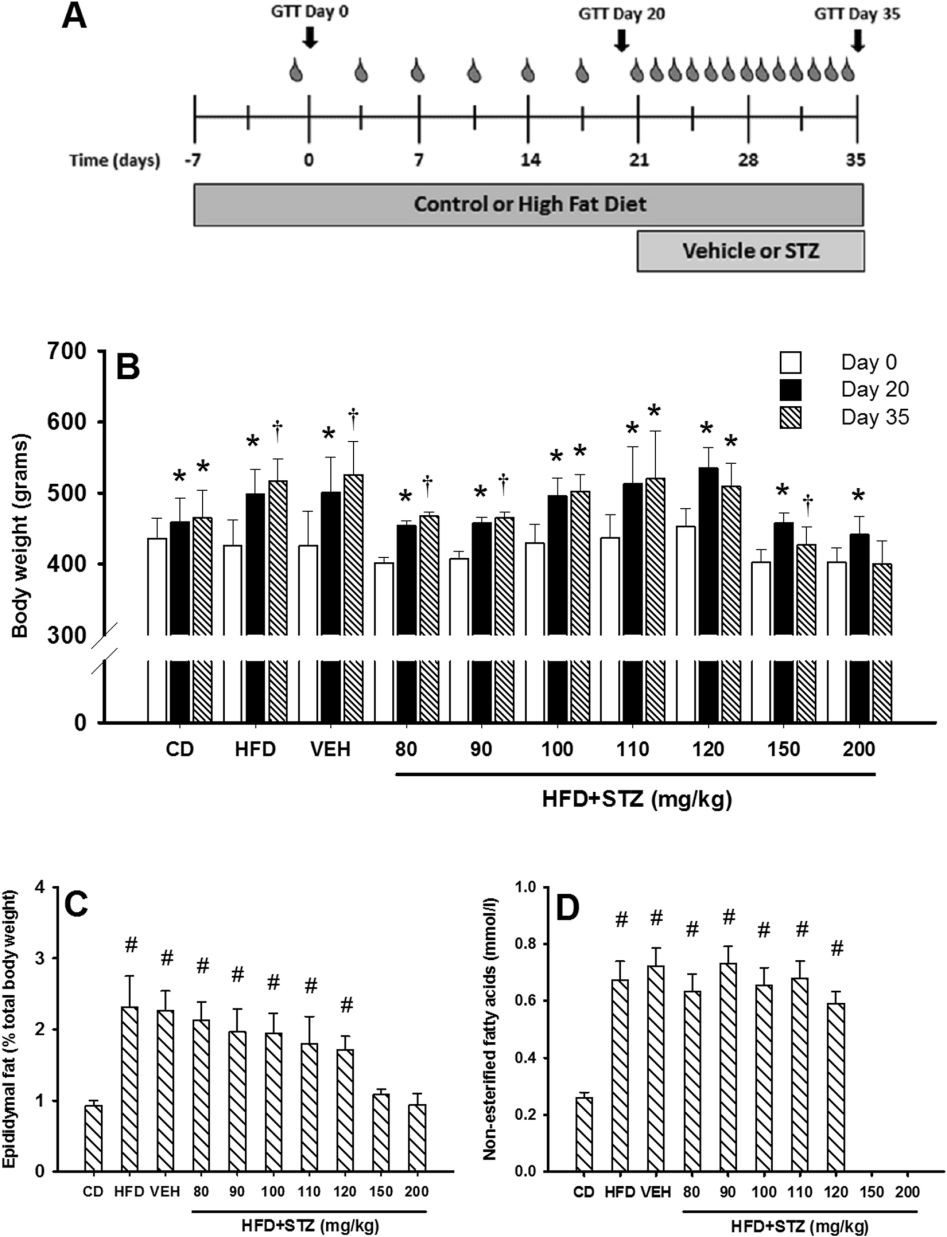
Osmotic mini-pump delivered STZ does not cause weight loss and animals retain an obesogenic phenotype. (**A**) Thirty-five day protocol for induction of type 2 diabetes using osmotic mini-pump infused STZ. Blood drops indicate assessment of body weight, food and water intake and non-fasting glucose concentrations. Glucose tolerance (1 g/kg; GTT) was assessed in each animal on days 0, 20 and 35. (**B**) Body weight plotted for day 0 (white bars), day 20 (closed bars) and day 35 (hatched bars). (**C**) Epididymal fat pads were excised and weighed at the end of the experiment (day 35) and presented as percent of total body weight to adjust for differences in total body weight across the groups. (**D**) Circulating non-esterified fatty acids were assessed on day 35 for all but the 150 and 200 mg/kg STZ groups where insufficient blood could be collected due to poor tail blood flow. Data are mean ± SEM for n = 6 in all groups except 150 mg/kg and 200 mg/kg STZ groups where n = 3. *p < 0.05 versus day 0 within the group; and ^†^p < 0.05 versus days 0 and 20 within the group using two-way repeated measures ANOVA. ^#^p < 0.05 versus CD using one-way ANOVA on ranks.

While water intake was not altered by HFD, food intake was greater in all HFD-fed groups relative to control diet (CD) across the first three weeks (Supplementary Table 1). In all groups, HFD feeding led to increased body weight compared CD-fed rats across the first three weeks and only the two highest doses of STZ (150 mg/kg and 200 mg/kg) led to weight loss at the end of the five-week period (Fig. 2B). Epididymal fat mass (a surrogate marker of adiposity) was approximately 2-fold higher in all HFD-fed animals compared to CD (p < 0.05 for all) with the exception of the 150 mg/kg and 200 mg/kg STZ groups (Fig. 2C), where there was no difference. Similarly, all HFD fed rats exhibited 2–3 fold higher circulating non-esterified fatty acid (NEFA) concentrations relative to CD (Fig. 2D; p < 0.001 for all).

### Osmotic mini-pump-infused STZ dose-dependently increased fasting glucose and decreased fasting insulin concentrations

Non-fasting glucose concentrations were assessed in all animals twice weekly up to day 20 and then daily from days 21–34 (Supplementary Figure 1). Six days post-mini pump implant, non-fasting glucose concentrations were increased, but not statistically different from vehicle at STZ doses ranging from 80–110 mg/kg. Animals that received 120 mg/kg STZ had increased non-fasting glucose by day five and this remained higher than vehicle across the reminder of the intervention (p < 0.01). The two highest doses of STZ tested caused a rapid and overt increase in non-fasting glucose concentrations 2–3 days following mini-pump implant. In both cases, glucose concentrations exceeded 20.0 mmol/l within 6–7 days’ post-implant. The experimental protocols in these two groups were concluded early (days 32 and 28, respectively) due to rapid weight loss (Fig. 2B) and animal health and wellbeing.

**Figure 3:**
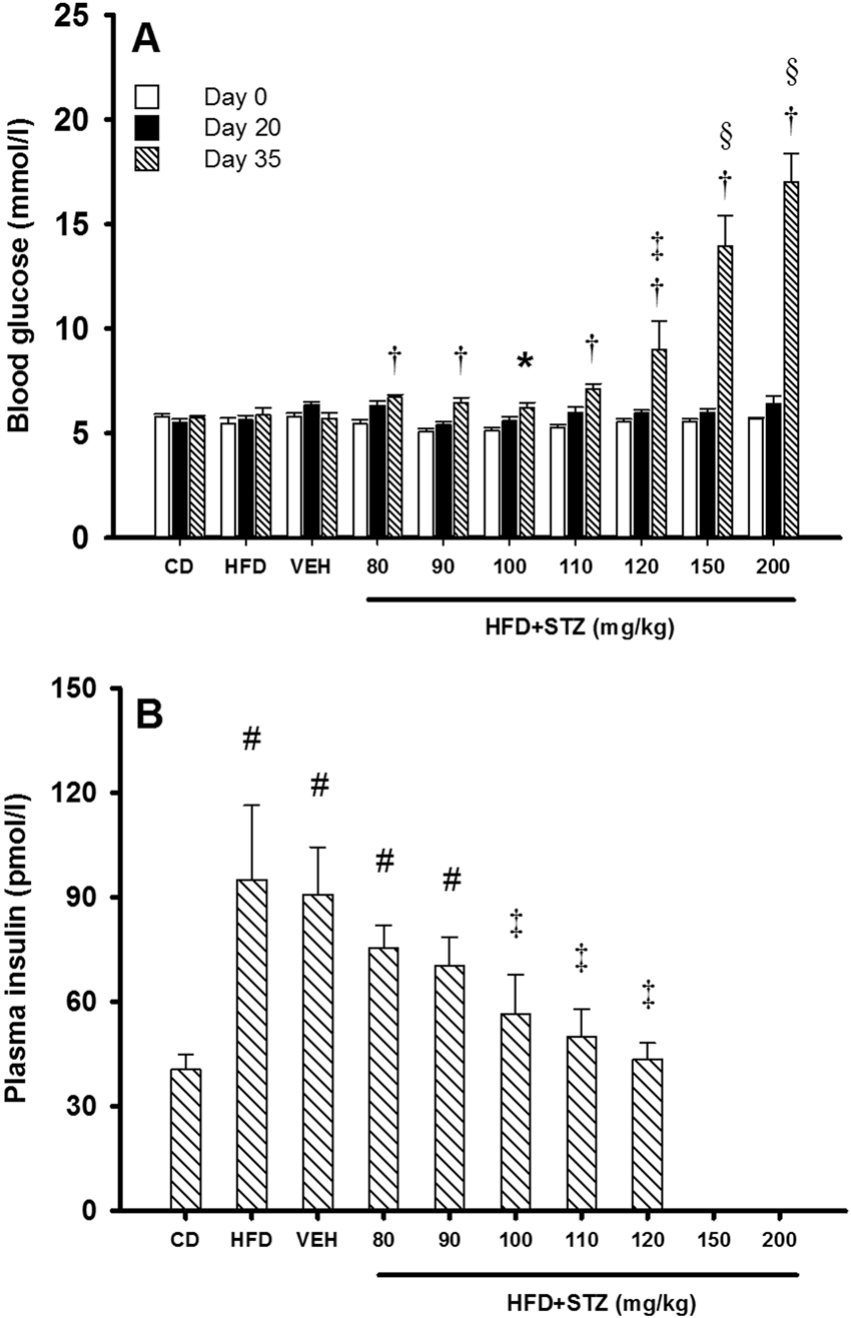
Osmotic mini-pump-infused STZ dose-dependently increased fasting glucose concentrations and reduced fasting insulin concentrations. (**A**) Fasting blood glucose concentrations were determined following an overnight fast on days 0 (open bars), 20 (closed bars) and 35 (hatched bars). The exception was 150 mg/kg and 200 mg/kg STZ groups where experiments ended on days 32 and 28 respectively for animal welfare. Data are mean ± SEM for n = 6 in all groups, except 150 mg/kg and 200 mg/kg STZ groups where n = 3. (**B**) On day 35, blood was collected prior to GTT and fasting plasma insulin determined. Data are mean ± SEM for n = 6 in all groups except 150 mg/kg and 200 mg/kg STZ groups where no samples could be collected due to poor tail blood flow. *p < 0.05 versus day 0 within the group; ^†^p < 0.05 versus days 0 and 20 within the group; ^§^p < 0.05 versus vehicle and the preceding dose of STZ at day 35 using two-way repeated measures ANOVA. ^#^p < 0.05 versus CD; and ^‡^p < 0.05 versus vehicle at day 35 using one-way ANOVA on ranks.

All animals were fasted overnight prior to glucose tolerance tests on days 0, 20 and 35. Fasting blood glucose concentrations were not different between groups at baseline or day 20 (Fig. 3A). On day 35, fasting glucose was increased in animals treated with 80 mg/kg STZ (6.7 ± 0.1 mmol/l), 90 mg/kg STZ (6.5 ± 0.3 mmol/l) and 100 mg/kg STZ (6.4 ± 0.3 mmol/l) compared with their respective values on day 0 and day 20 (p < 0.05 for all), but did not reach significance versus vehicle (5.7 ± 0.3 mmol/l). Relative to vehicle, fasting glucose was increased in animals that received 110 mg/kg STZ (7.2 ± 0.2 mmol/l) and 120 mg/kg STZ (9.0 ± 1.3 mmol/l). Increasing STZ further to 150 mg/kg and 200 mg/kg resulted in extreme increases in fasting glucose (p < 0.001 for both vs vehicle).

Compared with CD-fed animals (41 ± 4 pmol/l), fasting insulin concentrations (Fig. 3B) were significantly higher in HFD (95 ± 20 pmol/l), vehicle (91 ± 14 pmol/l), 80 mg/kg (75 ± 7 pmol/l) and 90 mg/kg (70 ± 8 pmol/l) STZ groups (p < 0.05 for all). In animals that received 100 mg/kg (57 ± 11 pmol/l), 110 mg/kg (50 ± 8 pmol/l) and 120 mg/kg (43 ± 5 pmol/l) doses of STZ, fasting insulin concentrations were lower relative to vehicle (p < 0.05 for all) but remained modestly higher than CD-fed rats. Unfortunately, due to poor blood circulation in the tails of animals that received 150 mg/kg and 200 mg/kg STZ, we could not collect enough blood to determine insulin concentrations in these animals, but based on the characteristics of these animals it is highly likely that both groups had very low circulating insulin.

### Osmotic mini-pump-infused STZ dose-dependently decreased glucose tolerance

**Figure 4:**
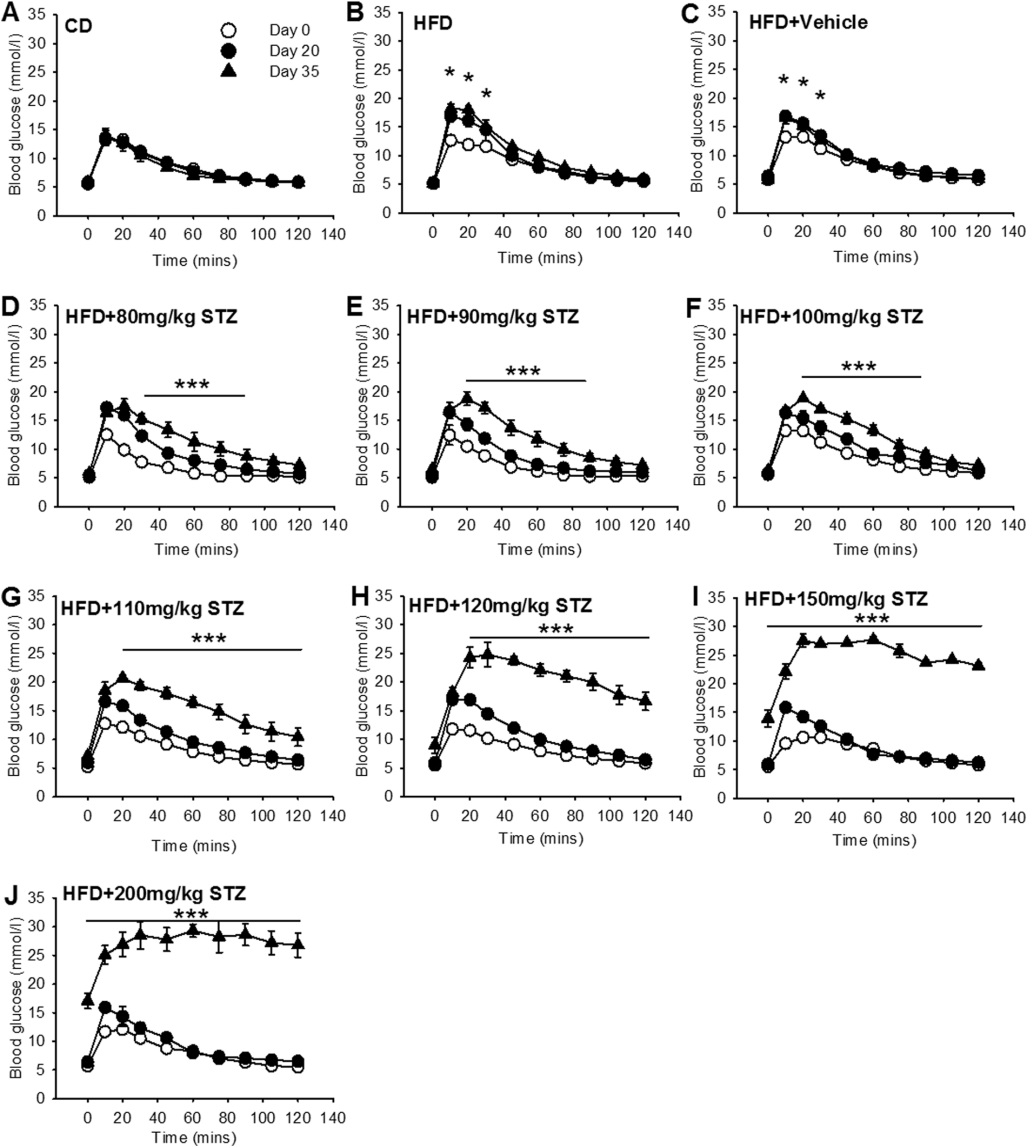
Osmotic mini-pump-infused STZ dose-dependently decreased glucose tolerance. Following an overnight fast, blood was sampled from the tip of the tail and circulating blood glucose concentrations were determined at 0, 10, 20, 30, 45, 60, 75, 90, 105 and 120 min after glucose injection (1 g/kg). Glucose tolerance tests were performed on day 0 (open circle), day 20 (closed circle) and day 35 (closed triangle) except 150 mg/kg and 200 mg/kg STZ groups where experiments ended on days 32 and 28 respectively. (**A**) CD; (**B**) HFD; (**C**) vehicle; (**D**) 80 mg/kg; (**E)** 90 mg/kg; (**F**) 100 mg/kg; (**G**) 110 mg/kg; and (**H**) 120 mg/kg; (**I**) 150 mg/kg; (**J**) 200 mg/kg STZ. Data are mean ± SEM for n = 6 in all groups except 150 mg/kg and 200 mg/kg STZ groups where n = 3. *p < 0.05 versus day 0 within the group; and ***p < 0.05 versus both days 0 and 20 at all time-points under the line using two-way repeated measures ANOVA.

**Figure 5:**
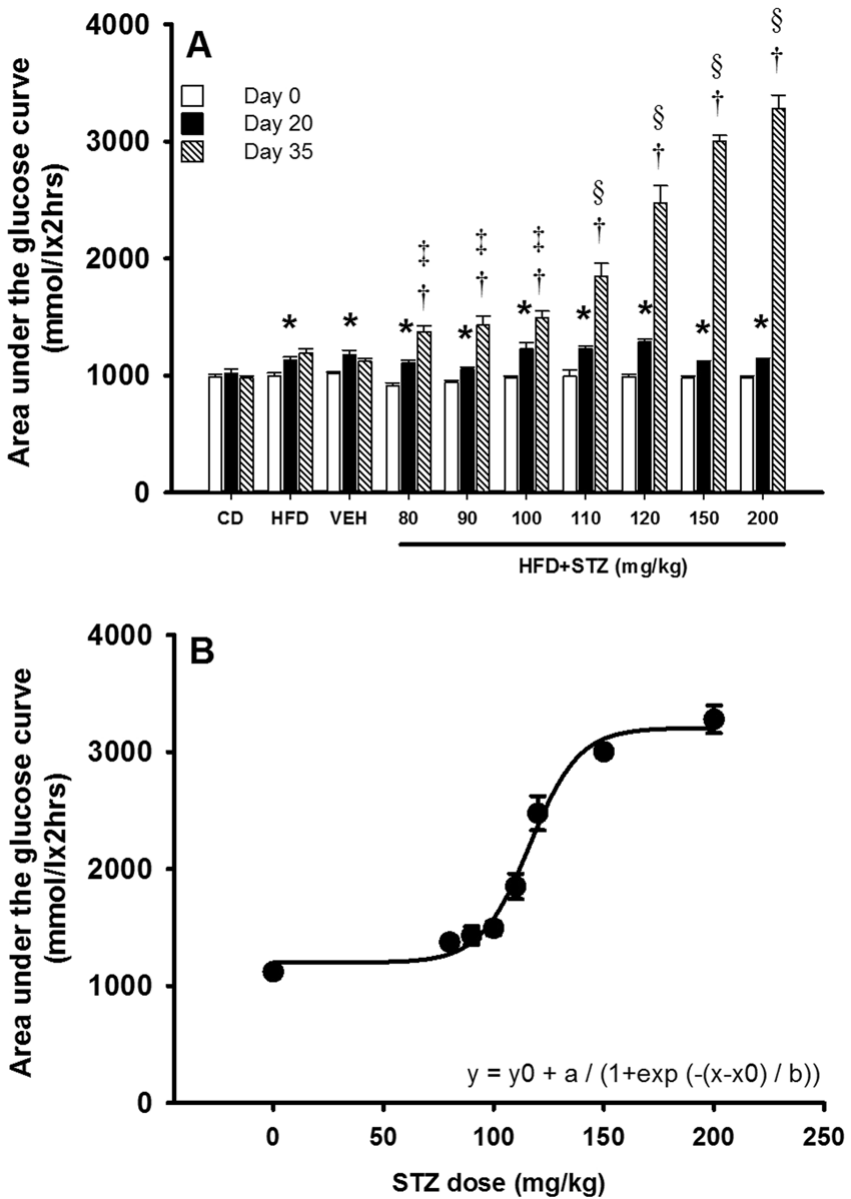
Osmotic mini-pump-infused STZ dose-dependently increased glucose exposure during a glucose tolerance test. (**A**) The area under the glucose curve (AUC) was calculated for glucose tolerance test at each of the three time-points (day 0 [open bars], day 20 [closed bars] and 35 [hatched bars]) using the trapezoidal method. (**B**) A dose-response curve was generated by plotting AUC measures versus STZ dose. Data are mean ± SEM for n = 6 in all groups except 150 mg/kg and 200 mg/kg STZ groups where n = 3. *p < 0.05 versus day 0 within the group; ^†^p < 0.05 versus days 0 and 20 within the group; ^‡^p < 0.05 versus vehicle at day 35; and ^§^p < 0.05 versus vehicle and the preceding dose of STZ at day 35 using two-way repeated measures ANOVA.

Glucose tolerance was assessed on day 0, day 20 and day 35 using a 1 g/kg i.p. injection of glucose (Fig. 4). The total exposure to glucose during the glucose tolerance test was quantified by calculating the area under the curve (AUC) for each test (Fig. 5A). On day 20 all HFD-fed rats exhibited significantly higher glucose concentrations across the first 30 min (Fig. 4B; p < 0.05), culminating in a significant increase in total glucose AUC relative to day 0 across all groups (Fig. 5A; p < 0.01). On day 35, glucose tolerance was not different between HFD and vehicle treated rats (Figs 4B,C and 5A). Animals that received 80–100 mg/kg STZ exhibited significantly increased glucose concentrations from 30–90 min (p < 0.05) but returned toward baseline over the final 30 min of the glucose tolerance test (Fig. 4D–F). All three STZ groups exhibited a marked increase in AUC during the glucose tolerance test relative to vehicle treated rats (Fig. 5A). Treatment with 110 mg/kg STZ caused a further decline in glucose tolerance and glucose concentrations remained significantly elevated relative to baseline across the time-course of the glucose tolerance test (Fig. 4G; p < 0.01). Increasing STZ further to 120 mg/kg, 150 mg/kg and 200 mg/kg resulted in step-wise increases in glucose concentrations across the 120 min test where there was minimal return baseline (Fig. 4H–J). These findings were mirrored by step-wise increases in glucose AUC during the glucose tolerance test for each dose of STZ (Fig. 5A; p < 0.001 vs vehicle and preceding STZ dose for all). Plotting the STZ dose versus the resulting AUC (Fig. 5B) revealed a tight sigmoidal, dose-dependent relationship where STZ appears to have small effects on glucose tolerance at 80 mg/kg and near maximal effects at 150 mg/kg.

### Osmotic mini-pump-infused STZ reduced insulin release during a glucose tolerance test

**Figure 6:**
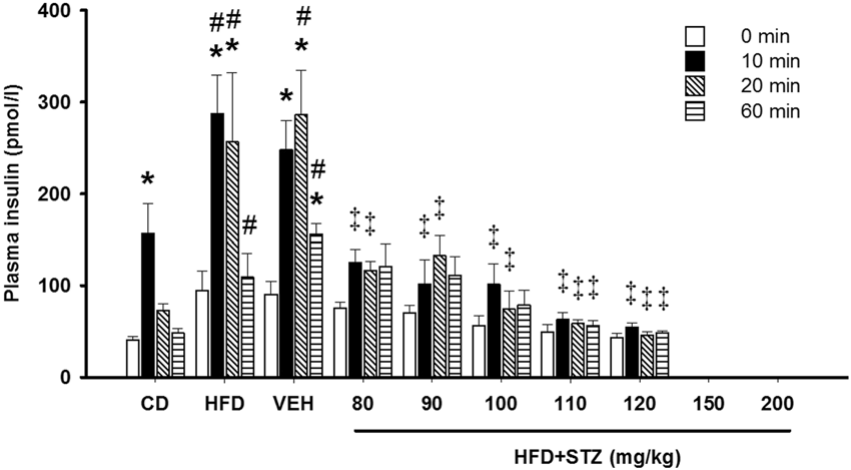
Osmotic mini-pump-infused STZ reduced insulin secretion during a glucose tolerance test. Blood was collected at 0 (open bars), 10 (closed bars), 20 (diagonal hatch) and 60 min (horizontal hatch) into the glucose tolerance test and insulin concentrations quantified. Data are mean ± SEM for n = 6 in all groups except 150 mg/kg and 200 mg/kg STZ groups where no samples could be collected due to poor tail blood flow. *p < 0.05 versus 0 min within the group; ^#^p < 0.05 versus CD at the same time-point; and ^‡^p < 0.05 versus vehicle at the same time-point using two-way repeated measures ANOVA.

Plasma was collected at 0, 10, 20 and 60 min during the day-35 glucose tolerance test (Fig. 6) in all groups with the exception of the 150 and 200 mg/kg STZ groups. In CD-fed animals, a significant rise in plasma insulin concentrations was detected 10 min following glucose injection (41 ± 4 vs. 155 ± 32 pmol/l; p < 0.001) but quickly returned toward baseline at 20 (73 ± 8 pmol/l; p = 0.482) and 60 min (48 ± 5 pmol/l; p = 0.786). Both the HFD-only and HFD+ vehicle treated groups had higher plasma insulin concentrations at 10 min (287 ± 42 and 249 ± 32 pmol/l), 20 min (257 ± 76 and 286 ± 49 pmol/l) and 60 min (110 ± 25 and 156 ± 12 pmol/l) compared with CD (p < 0.05 for all time-points). Animals that received 80 mg/kg STZ had modest increases in insulin concentrations at 10 min (125 ± 14 pmol/l; p = 0.291), 20 min (116 ± 10 pmol/l; p = 0.151) and 60 min (121 ± 25 pmol/l; p = 0.244) relative to their baseline. Similarly, at 90 mg/kg STZ, circulating insulin increased modestly following glucose injection at 10 min (102 ± 25 pmol/l; p = 0.265), 20 min (133 ± 22 pmol/l; p = 0.127) and 60 min (111 ± 20 pmol/l; p = 0.319) relative to bassline. At 100 mg/kg STZ, insulin concentrations were higher at 10 min (102 ± 23 pmol/l; p = 0.387), but returned toward baseline at 20 min (75 ± 19 pmol/l; p = 0.522) and 60 min (79 ± 16 pmol/l; p = 0.709). Relative to vehicle treated animals, the rats that received 80, 90 and 100 mg/kg STZ exhibited reduced insulin concentrations at 10 and 20 min following glucose administration (Fig. 6; p < 0.001 for all). Animals that received 110 mg/kg and 120 mg/kg STZ did not exhibit increased insulin levels at any time-points following glucose administration and had lower insulin concentrations relative to vehicle treated animals at all time-points (Fig. 6; p < 0.05 for all).

Pancreatic tissue was collected from each animal and representative sections are shown for each group (Supplementary Figure 2). Islets from CD, HFD and vehicle (panels A–C) treated animals had similar structure and stained heavily for insulin/pro-insulin. At STZ doses of 80–120 mg/kg, there appeared to be a step-wise decline in islet staining for insulin/pro-insulin, while islet structure shifted from oval toward irregular in shape (panels D–H). Very few islets were present in sections from 150 mg/kg and 200 mg/kg STZ groups (panels I and J) and when located, exhibited almost no staining for insulin/pro-insulin.

## Discussion

In the present study, we demonstrate a novel method that allows for targeted and sustained induction type 2 diabetes in rats by combining a HFD with osmotic mini-pump-infused STZ. In our model, HFD feeding led to obesity, increased circulating NEFA and insulin concentrations and glucose intolerance, typical of insulin resistance/pre-diabetes in both rodents^7,22^ and humans^23^. The addition of STZ after three weeks of HFD feeding caused a dose-dependent increase in circulating glucose and further decline in glucose tolerance while preserving an obese, dyslipidemic phenotype. Critically, the dose-dependent decline in glucose tolerance was accompanied by a reciprocal, dose-dependent decrease in insulin secretion in response to the glucose load. Thus, for the first time our novel approach enables targeted induction of type 2 diabetes across the spectrum of disease. This approach is a significant advance for modelling distinct stages of type 2 diabetes (i.e. during the phase of declining beta cell function) that ultimately provides a powerful platform for modelling type 2 diabetes and associated pathologies.

Once in the circulation, STZ is transported into pancreatic beta cells by the glucose transporter 2 (GLUT2) protein^24,25^. Within the beta cell, STZ interrupts a number of important cellular processes and if the insult is sufficient, culminates in DNA damage and cell death^24,25^. The end result of STZ administration is the reduction of functional beta cell mass that manifests as insulin deficiency and subsequent inability to handle glucose^25^. Coupling this insulin insufficiency with the challenge of a HFD, where increased insulin is required to account for cellular insulin resistance^26,27^, induces a state of glucose intolerance^8^, typical of human type 2 diabetes. In the present study, we demonstrate that the combination of HFD + STZ infused by osmotic mini-pump enables excellent phenotypic control with targeted induction of hyperglycemia and glucose intolerance, while maintaining a state of obesity. Furthermore, we confirmed that the dose-dependent effect of STZ we observed is due to a reciprocal reduction in insulin secretory capacity and morphological changes to the pancreas. To our knowledge, no other method currently allows such precise control over the resulting level of hyperglycaemia and glucose intolerance. Therefore, we believe this is the first animal model capable of reproducing different stages of type 2 diabetes defined by dose-dependent effects of STZ on glucose intolerance, reduced beta cell function and ensuing hyperglycemia while retaining an obese dyslipidemic profile.

All current methods for delivering STZ employ injections (e.g. IP or IV). Whilst these are exclusively utilised in the literature (due to the prevailing notion that STZ degrades quickly in solution), injection methods also impose significant limitations to the resulting phenotype. IP injections yield highly variable results due to the exact injection site and thus influences the absorbance rate of STZ into the systemic circulation. This may account for some of the inconsistencies in the literature where similar doses of STZ (e.g. 35 mg/kg) are reported to have very different effects on glucose tolerance^8,28,29^. Similar issues confound studies using IV injection to administer STZ, where at the same dose as IP (35 mg/kg), IV injections are reported to be just as variable in their effectiveness^30,31^. Generally, the IV method appears to be preferred, likely due to a more targeted and direct mode of delivery^15^. However, relative to IP, IV injections are more technically challenging without providing significant improvement over the resulting level of hyperglycemia. A recent study in guinea pigs demonstrated that subcutaneous injection of STZ (200 mg/kg) could improve overall efficacy but only when animals are also treated with the α-adrenergic receptor antagonist yohimbine^12^. In the present study, we show consistent, reproducible and dose-dependent effects by utilising subcutaneously implanted osmotic mini-pumps to infuse STZ over the course of 14 days.

Injection methods cause spikes in circulating STZ concentrations and as such, STZ has a very short time-frame to affect pancreatic beta cells. Whether this short, single-pass insult to the beta cells is sufficient to induce cell death is highly dependent on the dose of STZ utilised. Indeed, animals injected with lower STZ doses (15–30 mg/kg IP or IV) have been reported to revert back to normoglycemia, and thus require repeat low-dose injections to maintain the hyperglycaemic phenotype^18^. Conversely, animals injected with higher STZ doses (≥50 mg/kg IP or IV) exhibit overt hyperglycemia, complete absence of insulin, weight loss and as such are prone to a high mortality rate^15,18,32^. In support of one previous study^33^ and in contrast the prevailing belief that STZ degrades quickly, we report that STZ undergoes an isomer switch and remains relatively stable for at least 14 days. This opens the door to novel avenues of STZ administration, such as the osmotic mini-pump, where STZ can be released slowly and consistently into the circulation over 14 days. Therefore, rather than a large spike in STZ, cells are exposed to low circulating concentrations of STZ resulting in a sustained 14-day insult. Importantly, even though circulating STZ concentration is relatively low compared to injection methods, the sensitivity of beta cells to STZ-induced damage is evident from the rapid and sustained effects on glucose handling, circulating insulin and pancreatic histology. Thus, we conclude that the overall improvement in effectiveness observed in our study is likely due to sustained, chronic exposure of STZ to beta cells. Our data suggest that a sustained low-grade insult to beta cells produces more consistent and precise effects than that of a large dose with single-pass uptake. Therefore, administering STZ using osmotic mini-pumps imparts significantly greater control over the resulting level of hyperglycemia while retaining an obesogenic phenotype.

From an animal welfare perspective and in accordance with the NC3Rs and the ARRIVE guidelines for animal research^34^, our effective STZ dose range of 80–120 mg/kg led to consistent metabolic effects in all animals (zero non-responders) and resulted in zero inadvertent animal deaths. While these statistics are generally poorly reported in the literature as a whole, our method is a marked improvement on previous publications where animals either do not respond to STZ injection (~10–15%) or die as a result of STZ injection (≥30%)^15,33^. Taken together, these data demonstrate that our new method required fewer animals to observe significant effects compared with previous reports^15,33^. As noted above, the improvement in efficacy that we observed is likely due to the prevailing concentration of STZ in the circulation following injection (very high) or osmotic mini-pump infusion (very low) and the resulting effects on not just the highly sensitive beta cells, but also all other cells in the body.

It should be noted that while our method is a significant advance in the field, the HFD + STZ model itself remains somewhat artificial with regard to human type 2 diabetes pathogenesis. Indeed, individuals with type 2 diabetes progress through a far longer stage of insulin resistance-associated hyperinsulinemia, which ultimately leads to beta cell dysfunction^5,6^. While our model involves a background of HFD-induced insulin resistance^7,22^, the administration of STZ does not replicate the exact cause of beta cell death as seen in humans. While this imposes some limitations on this model, the use of STZ greatly accelerates the time-frame for development of hyperglycemia and glucose intolerance that may not otherwise occur in rodents with relatively short life-spans. Importantly, the result of both human type 2 diabetes and STZ administration is the same, development of sustained hyperglycemia, which ultimately drives the pathological features of type 2 diabetes such as microvascular disease, neuropathies and nephropathy.

In conclusion, our new approach provides a significant advance and enables induction of distinct stages of clinical type 2 diabetes in rodents. Ultimately, this new model provides a powerful platform for identification of preventative and therapeutic strategies targeting the spectrum of type 2 diabetes and associated pathologies.

## Materials and Methods

### Stability of STZ across 14 days

The stability of STZ over the 14 day delivery period of the osmotic mini-pump was determined using reverse-phase high performance liquid chromatography (HPLC) as previously described^33^. Briefly, four independent STZ solutions (3.5 mg/ml) were prepared in citrate buffered saline (pH 4.4) and kept in the dark at 37 °C for 14 days. STZ (40 µl) was injected into a C18 column (5 µm, Gemini, Phenomenex, CA, USA) and analysed under isocratic conditions using a flow rate of 1.0 ml/min with a 3% methanol/97% acetate buffer (pH 4.4) and detection at a wavelength of 250 nm. To investigate the α and β STZ anomer equilibration dynamics, each solution was sampled at 0, 20, 40, 60, 80 and 100 min after dissolution. To investigate STZ degradation across 14 days, each solution was subsequently sampled every two days after dissolution.

### Animal husbandry

All experimental procedures were approved by the University of Tasmania Animal Ethics Committee and performed in accordance with the Australian Code for the Care and Use of Animals for Scientific Purposes – 2013, 8^th^ Edition. Male Sprague Dawley rats aged 14–16 weeks were obtained from the University of Tasmania animal facility or Monash Animal Services and allowed to acclimatise for 7 days upon arrival. Rats were randomised and housed in groups of three at 21 ± 2 °C with a 12 hr-12 hr light-dark cycle. All animals were provided with water and chow *ad libitum* for throughout the study. With the exception of control diet (CD) only fed rats, all other groups were provided with a high fat diet (HFD; 23% fat by weight; simple carbohydrate replacement; Specialty Feeds, WA, Australia) *ad libitum* throughout the study (see Fig. 2A).

### Osmotic mini-pump implantation and STZ dosage

Following three weeks of HFD, STZ (Sigma Aldrich, St Louis, MO, USA) was infused at a constant rate (5 µL/hr) over a 14-day period using a subcutaneously implanted osmotic mini-pump (Alzet Model 2ML2; Durect Corporation, Cupertino, CA, USA). Briefly, rat anesthesia was maintained using isoflurane throughout the surgical procedure. Following sterile preparation of the surgical site, a 1.5 cm incision (perpendicular to the spine) was made through the skin on the dorsum of the animals at the lumbar level of the spinal cord. Blunt dissection was used to separate connective tissue between the skin and underlying muscle layers to allow for insertion of the osmotic mini-pump. Loaded mini-pumps were inserted and positioned inside the cavity between the scapulae so that delivery of solution occurred away from the surgical site. The incision site was sealed using dissolvable sutures and animals allowed to recover.

There are no previous reports of STZ delivery using osmotic mini-pumps. Due to the slow flow rate of the osmotic mini-pumps, we tested seven different STZ concentrations to establish an STZ dose-response curve. STZ was prepared in citrate buffered saline (0.1 mmol/l; pH 4.4) and a total volume of 2 ml was loaded into mini-pumps 20–30 min prior to surgical implant. The seven STZ doses we tested were; i) 200 mg/kg; ii) 150 mg/kg; iii) 120 mg/kg; iv) 110 mg/kg; v) 100 mg/kg; vi) 90 mg/kg; and vii) 80 mg/kg (these doses represent the total amount of STZ delivered to the animal over 14 days). Given the flow rate of the 2ML2 pump (5 µl/hr) and assuming all animals weighed 500 g on the day of implant, this would equate to STZ doses of; i) 0.25 mg/hr; ii) 0.19 mg/hr; iii) 0.15 mg/hr; iv) 0.14 mg/hr; v) 0.13 mg/hr; vi) 0.11 mg/hr; and vii) 0.10 mg/hr over a 14-day period. These equated to a dose-range of 2.4–6 mg/day, far lower than the 20–100 mg/kg doses used typically in IP or IV experiments^8,28–31^. Vehicle treated rats received mini-pumps containing citrate buffered saline (pH 4.4; 2 ml). To control for any diet-specific effects, two additional groups of rats were maintained on CD or HFD only for the duration of the 35-day protocol (Fig. 1). Thus, a total of 10 groups were used in the present study.

### Assessment of metabolic alterations

Food intake, water intake, body weight and non-fasting blood glucose concentrations were assessed every 3–4 days for the duration of the 35-day intervention (see Fig. 2A). Non-fasting glucose concentrations were determined using a hand-held glucometer (Accu-Chek Performa, Roche Diagnostics, NSW, Australia) by making a small incision to the tip of the tail to access blood. To assess changes in glucose handling over the 35-day intervention each animal underwent a glucose tolerance test at three distinct time-points; i) Day 0 - baseline; ii) Day 20 – HFD-induced insulin resistance; and iii) Day 35 – following STZ or vehicle delivery.

All glucose tolerance tests were performed on overnight fasted rats. Briefly, animals received an intraperitoneal injection of glucose (1 g/kg). Blood was accessed from the tip of the tail and glucose concentrations were measured at 0, 10, 20, 30, 45, 60, 75, 90, 105 and 120 min following glucose injection. During the day 35 glucose tolerance test, larger volumes of blood (2–3 drops) were collected at 0, 10, 20 and 60 min following glucose injection in pre-heparinised tubes, centrifuged and plasma stored at −20 °C. Plasma insulin (Mercodia Rat Insulin ELISA, Uppsala, Sweden) and non-esterified fatty acid concentrations (NEFA-C; Wako Pure Chemical Industries, Osaka, Japan) were determined according to the manufacturer’s instructions.

### Data and statistics

All data generated or analysed during this study are included in this published article (and its Supplementary Information files). Statistics and graphical presentation of data was carried out using Sigma Plot 11 (Systat Software, San Jose, CAL, USA). In instances where data was not normally distributed, Kruskal-Wallis one way ANOVA on Ranks was utilised with Student-Newman-Keul’s post-hoc. Where data was normally distributed, one-way ANOVA with Student-Newman-Keul’s post-hoc was group differences at a single time-point and two-way repeated measures ANOVA with Student-Newman-Keul’s post-hoc was utilised for all time-course comparisons. A value of *p* < 0.05 was considered statistically significant.

## Electronic supplementary material

Supplementary Methods and Results
