# Supplementary material for: A New Method for Targeted and Sustained Induction of Type 2 Diabetes in Rodents
Source: Sci Rep. 2017 Oct 26;7:14158. doi: 10.1038/s41598-017-14114-4 (PMC5658444; doi:10.1038/s41598-017-14114-4)
Supplement: Supplementary file 1 — Supplementary Methods and Results [file 41598_2017_14114_MOESM1_ESM.pdf]

**SUPPLEMENTARY INFORMATION**

**TITLE: A New Method for Targeted and Sustained Induction of Type 2 Diabetes in Rodents.**

**AUTHORS: Dino Premilovac[1], Robert Gasperini[1], Sarah Sawyer[1], Adrian West[1], Michelle A Keske[2,3], Bruce V Taylor[2] and Lisa Foa[1].**

**Supplementary Methods:**

*Pancreas Histology*

All animals were euthanized using carbon dioxide and the pancreas of each animal was immediately excised and fixed overnight using neutral buffered formalin (4% formaldehyde; Dako, Glustrup, Denmark). The following day, samples were rinsed in PBS for 60min, transferred to 70% ethanol and processed for paraffin wax embedding using a Leica Autoprocessor (Leica Biosystems, Nussloch, Germany). Pancreas samples were embedded in paraffin and 14µm sections collected, transferred to slides and left to dry overnight at 35°C. Sections were deparaffinised, rehydrated and heat-induced epitope retrieval was carried out in citrate buffer (pH 6.0) using a pressure cooker for 20 min. After cooling, sections were blocked for 60 min at room temperature with 5% goat serum (Sigma Aldrich) in 50 mM Tris-buffered saline (TBS; pH 7.6) containing 0.4% triton X-100 (Sigma Aldrich). Sections were incubated overnight at 4°C in primary antibody: rabbit anti-insulin/pro-insulin (1:1000; Catalogue number ab8304; Abcam, Cambridge, United Kingdom). The following day, endogenous peroxidase activity was blocked using 0.3% $H_2O_2$ (Sigma Aldrich) for 15 min at room temperature. Subsequently, sections were probed with horseradish peroxidase linked goat anti-rabbit secondary antibody (1:1000; Dako) for 60 min at room temperature. Finally, sections were incubated with 3,3'-diaminobenzidine (DAB; Dako) for 15 min at room temperature as per the manufacturer's instruction. Counterstaining was performed using haematoxylin (Meyers Haematoxylin; Australian Biostain, NSW, Australia) and coverslips were mounted using DPX neutral mounting media (Koch-Light

Laboratories, Suffolk, United Kingdom). Images were captured using a Leica DM2500 microscope and Leica DFC495 camera (Leica Biosystems).

**Supplementary Table 1.**

| | **Food intake (calories/day)** | | | **Water intake (ml/day)** | | |
|---|---|---|---|---|---|---|
| | Week 1 | Week 3 | Week 5 | Week 1 | Week 3 | Week 5 |
| CD | 95±5 | 86±2 | 88±4 | 30±5 | 27±4 | 24±2 |
| HFD | 128±12# | 106±17# | 82±4*# | 23±4 | 23±4 | 19±1 |
| HFD + Vehicle | 148±9# | 94±3* | 82±8* | 24±4 | 20±4 | 21±6 |
| HFD + 80 mg/kg STZ | 128±15# | 88±3* | 78±4* | 22±3 | 20±1 | 18±2 |
| HFD + 90 mg/kg STZ | 119±10# | 90±5* | 80±4* | 25±4 | 20±1 | 23±2 |
| HFD + 100 mg/kg STZ | 146±8# | 89±8* | 81±7* | 25±3 | 20±2 | 20±7 |
| HFD + 110 mg/kg STZ | 128±7# | 97±6* | 84±7* | 21±1 | 20±1 | 21±1 |
| HFD + 120 mg/kg STZ | 130±6# | 91±6* | 79±4† | 23±3 | 20±2 | 47±21† |
| HFD + 150 mg/kg STZ | 103±15 | 94±1* | 80±6† | 27±5 | 25±3 | 69±14† |
| HFD + 200 mg/kg STZ | 104±2*# | 85±7* | 40±4† | 23±2 | 19±3 | 38±2† |

**Effect of osmotic mini-pump delivered STZ on food and water intake.** Food and water intake were assessed every 3-4 days from the start of the protocol. Data are means±SEM for n=6 in all groups except 150 mg/kg and 200 mg/kg STZ groups where n=3. * $p<0.05$ versus day 0 within the group; # $p<0.05$ versus CD at same time-point; † $p<0.05$ versus day 0 and day 20 within the group.

**Supplementary Figure 1.**

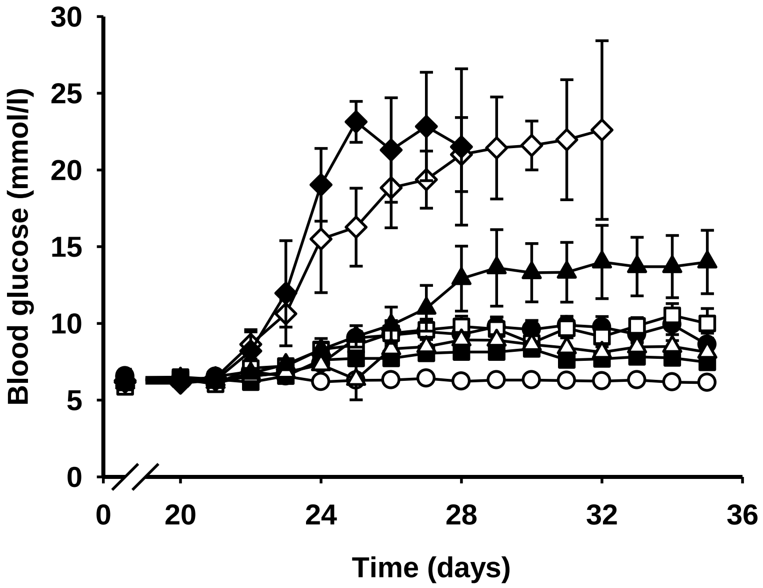


**Osmotic mini-pump-infused STZ increased non-fasting blood glucose concentrations.**
Daily non-fasting blood glucose concentrations are shown for vehicle (open circle), 80 mg/kg (closed circle), 90 mg/kg (open square), 100 mg/kg (closed square), 110 mg/kg (open triangle), 120 mg/kg (closed triangle), 150 mg/kg (open diamond) and 200 mg/kg STZ (closed diamond). Data are mean±SEM for n=6 in all groups except 150 mg/kg and 200 mg/kg STZ groups where n=3.

**Supplementary Figure 2.**

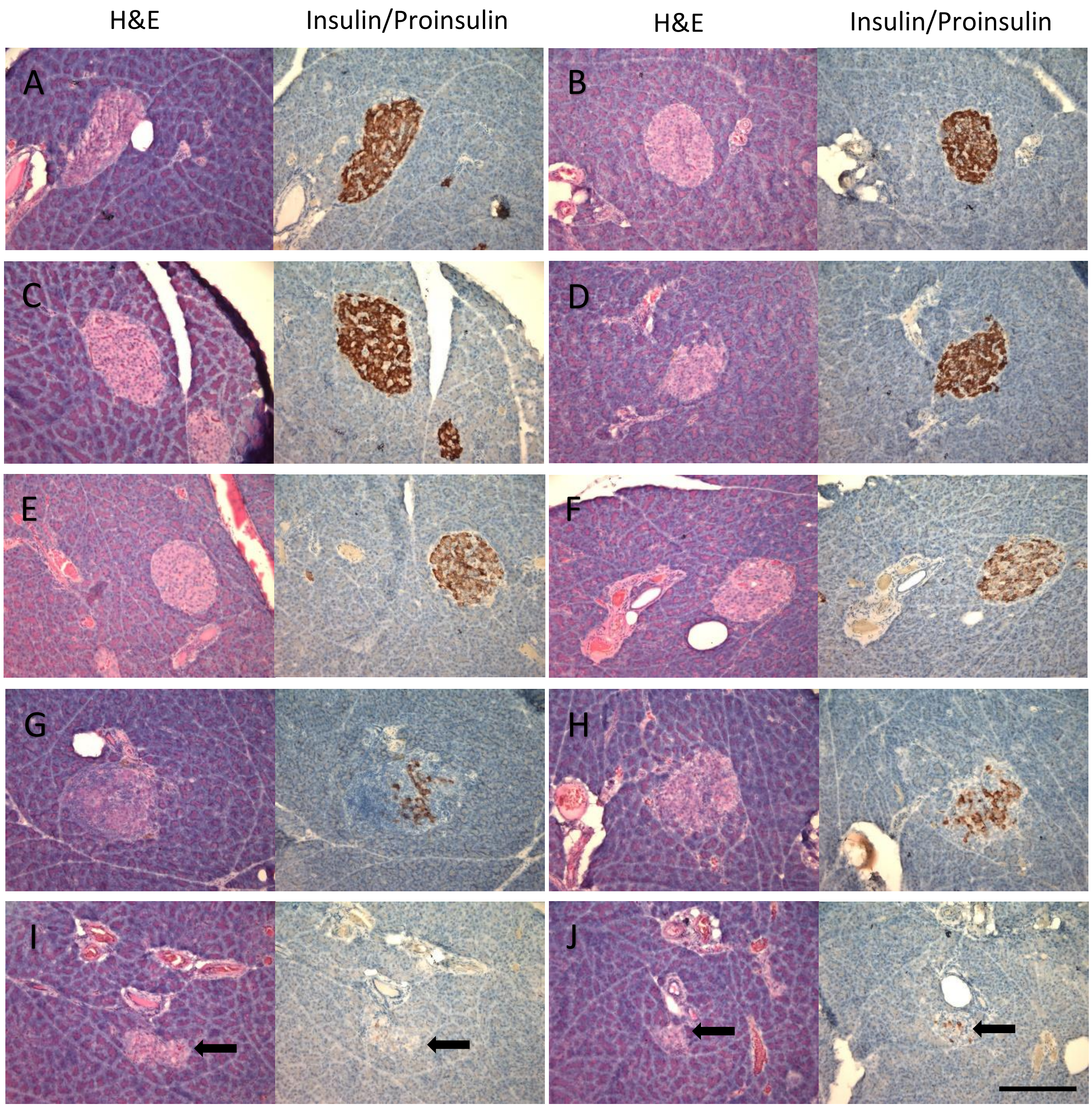


**Osmotic mini-pump-infused STZ decreases insulin positive staining within the Islets of Langerhans.** Sections were stained with either haematoxylin and eosin (H&E) to visualise islets or probed with a primary antibody for insulin/proinsulin, exposed to DAB and counterstained with haematoxylin. Representative images are shown for **A:** CD; **B:** HFD; **C:** Vehicle; **D:** 80 mg/kg; **E:** 90 mg/kg; **F:** 100 mg/kg: **G:** 110 mg/kg; **H:** 120 mg/kg; **I:** 150 mg/kg; and **J:** 200 mg/kg STZ. Arrows in panels I and J indicate Islets of Langerhans. Scale bar = 200μm.
